# A retrospective qualitative evaluation of barriers and facilitators to the implementation of a school-based running programme

**DOI:** 10.1186/s12889-018-6078-1

**Published:** 2018-10-20

**Authors:** Anna E. Chalkley, Ash C. Routen, Jo P. Harris, Lorraine A. Cale, Trish Gorely, Lauren B. Sherar

**Affiliations:** 1 National Centre of Sport and Exercise Medicine, School of Sport, Exercise and Health Sciences, Loughborough University, Loughborough, UK; 2 Department of Nursing, University of the Highlands and Islands, Inverness, Scotland, UK

**Keywords:** Implementation, Primary schools, Children, Physical activity, Qualitative

## Abstract

**Background:**

There is growing interest in school-based interventions which deliver opportunities for additional physical activity time outside of physical education (PE). A practical and cost-effective approach may be school running programmes. Consequently, many school-based running initiatives are currently being implemented in a grass-roots style movement across the UK. However, research on the implementation of physical activity programmes in schools is notably underdeveloped. Therefore, this qualitative study aimed to better understand the barriers and facilitators to the implementation of a running programme, Marathon Kids (MK), within primary schools in England.

**Methods:**

Two sets of semi-structured interviews were conducted, the first with each of the three core members of staff responsible for MK, and the second with each of the MK school staff Champions from 20 primary schools. Also, nine focus groups were conducted with 55 pupils (6–10 years) from five of the schools; all were analysed using thematic analysis.

**Results:**

Three themes were identified surrounding the barriers and facilitators to implementation: features of the programme (e.g. ethos and resources), school climate (e.g. culture; whole school engagement; PE and physical activity policies and goals; and physical environment) and programme implementation decisions (e.g. aspirations and planning and sustainability).

**Conclusion:**

Findings suggest that the barriers and facilitators to implementation are wide-ranging and include programme, organisational and system-level factors. Collectively pointing towards the need for a preparation period before implementation to understand schools’ readiness to implement and context-specific factors, both regarding organisational capacity and programme specific capacity.

**Electronic supplementary material:**

The online version of this article (10.1186/s12889-018-6078-1) contains supplementary material, which is available to authorized users.

## Background

Schools have long been seen as critical settings for the delivery of programmes to help children develop the knowledge, skills and confidence to lead a physically active lifestyle [1]. Indeed, the number of physical activity programmes available to schools has grown substantially in recent years [2]. The importance of using evidence-based interventions is widely recognised [3], and professional bodies such as the Society of Health and Physical Educators in America and the Association for Physical Education in England advocate their use [4, 5].

Many published school-based interventions have targeted physical activity within the context of physical education (PE) [2]. However, as curriculum pressures limit time dedicated to PE in schools [6] and the utility of physical activity to impact on educational as well as health outcomes is increasingly recognised [7], there is growing interest in interventions which provide opportunities for physical activity across all areas of the school day [8] such as recess [9], active lessons [10] and classroom energisers [11]. Indeed, the 2016 UK Government’s Childhood Obesity strategy [12] suggested schools should provide at least 30 min of moderate-to-vigorous physical activity per day. Furthermore, the strategy identified school-based running programmes as an approach to help achieve this ambition. Whilst encouraging, a school’s decision as to whether to, adopt a programme and if so, which, is heavily influenced by well-marketed programmes which are often compatible with what has previously been delivered in school [13]. As a consequence, there are now examples of physical activity based ‘innovations’ which, despite limited evidence of their efficacy and effectiveness, display very high rates of spontaneous uptake [14].

School running programmes have been part of comprehensive school health programmes in America for many years (e.g. Let’s Move! Active Schools [15]) and many school-based running initiatives (e.g. The Daily Mile [16]), are now being implemented across Europe, including the UK, in a ‘grass roots’ style movement [17]. With increased funding available in England for primary PE, school sport and physical activity via the school Sport Premium [18] and the Healthy Pupils Capital Fund [19], more and more schools will arguably be adopting or considering adopting such programmes.

School running programmes could provide schools with a simple, cost-effective and practical means of providing an opportunity for pupils to engage in non-competitive physical activity during the school day. Also, preliminary evidence suggests that school running programmes have the potential to improve children’s levels of physical activity, sedentary behaviour, fitness, and on-task behaviour [20].

Marathon Kids (MK) [21] is a primary school-based running programme, developed and implemented nationally since 2013 in the UK, by the charity Kids Run Free (KRF). MK has traditionally been promoted to schools via social media and parents and families attending KRF’s community running events (Kids Run Free in the Parks). MK, is distinct from other running programmes in the UK and worldwide as it challenges children to complete the equivalent distance of a marathon over a whole school year by running or walking laps of a course once or twice a week during their lunch break. Pupils receive a band for every lap completed which are recorded centrally within the school via a digital tracking system (DTS). Distance is accumulated and monitored over time and rewards are given at certain milestones, e.g. quarter, half, three quarter and full marathon.

Some strategies and tools are employed within the programme to assist with its implementation in school; these include the appointment of a Marathon Champion (i.e. a member of school staff who takes responsibility for co-ordinating MK) and a school launch. The school launch affords the opportunity to receive on-site support by a member of staff from KRF who delivers a marathon-themed assembly, measures and marks the running route(s), populates the DTS with pupils’ details, and provides training on the administration of the programme with the Marathon Champion and a selection of pupil peer leaders or Marathon Ambassadors. The finale of the school launch is the introduction of the pupils to the route and the opportunity to start collecting lap bands. There have been no effectiveness or implementation evaluations conducted on the programme to date.

While schools are provided with some guidance and recommendations for the delivery of MK; it can be delivered flexibly to suit the needs and circumstances of the school. Therefore, the implementation of MK, particularly regarding duration, frequency, timing and distance of running, is likely to vary significantly across schools. A 2015 systematic review of factors associated with the implementation of school physical activity programmes [22] found 22 unique implementation issues including time, resource availability/quality and supportive school climate. A 2018 review [23] also identified resource availability as well as the availability of staff as being important, alongside social influence from school management/boards and necessary teacher skills. However, the unique features of programmes mean that while common barriers and facilitators exist there will likely be implementation issues that are wholly unique to school running programmes. To the authors’ knowledge, there have been no studies to date examining the implementation of school running programmes.

The key factors underlying the successful implementation of MK, including how to maximise school engagement and which features of the programme are successful in influencing physical activity, are not yet known. Understanding how the constituents of MK can be best implemented is essential to ensure that the programme is reaching or has the potential to reach those schools and children who are most in need (i.e. the inactive) and for the health benefits to be realised. Such understanding may also importantly apply to the implementation of other school running programmes which follow a similar premise (i.e. The Daily Mile, The Golden Mile etc.).

## Methods

### Aim

The purpose of this retrospective qualitative investigation was to understand the barriers and facilitators to the implementation of the Marathon Kids running programme within primary schools in England.

### Sample selection, recruitment and ethics

Ethical approval was granted by Loughborough University Ethics Approvals (Human participants) Sub-Committee (R16-PO32). All participants provided prior written informed consent and were informed they could withdraw at any time without negative consequence.

As advocated by Creswell (2001), a purposive sampling approach was used to yield information-rich cases relating to the implementation of MK in practice [24]. This involved engaging with and recruiting a number of different stakeholders knowledgeable about and experienced with, the programme.

At the time of recruitment, three members of staff were responsible for delivering and or managing the programme at KRF. All three members were invited to take part in the study by way of a letter and information sheet, and all gave their consent to participate in the study.

Fifty four schools had registered for MK since the initial pilot in 2013, and the present data collection (2016) and all were eligible to participate in the study, regardless of the duration of implementation. Schools were initially approached via an email to the Head teacher from the MK Project Manager, with a letter describing the general purpose of the study and requesting confirmation of the contact details of the Marathon Champion within the school. A follow-up email was sent to the Head teacher, shortly followed by an email directly to the Marathon Champion, with a letter describing the general purpose of the study and proposing a phone call so that the study could be explained in more detail. All schools received a maximum of five contacts (two by email to the Head teacher, two by email to the Marathon Champion and a phone call to the school administrator) inviting them to participate. Recruitment took place over a period of 8 weeks.

### Procedure

A qualitative approach was used to collect rich data [25] and understand the diversity and complexity associated with the implementation of MK. Qualitative methodology, in particular, is useful for exploring barriers and facilitators to implementation [26] and allows programmes to be studied in detail [27]. The study methods and reporting have been completed in accordance with the Standards for Reporting Qualitative Research Checklist [28], see Additional file 1.

### Semi-structured interviews

All interviews were conducted by the first author, a White British female who is a doctoral student with 15 years of experience working in the children’s physical activity programming sector, and who has undergone external training in qualitative interview and focus group techniques.

Two sets of semi-structured interviews were conducted as part of the study. The first set consisted of face to face interviews with each of the three core members of staff responsible for MK at KRF (the Project Manager, Project Officer, and Founder and Chief Executive (CE) of KRF). The staff members were interviewed in a quiet private room at their offices.

A topic guide (see Additional file 2) was used to direct the interviews with probes and questions based on the following themes: roles and responsibilities in the delivery of MK; process of and variation in delivery of MK (by both KRF staff in launching the programme and school staff in implementing the programme); school, teacher and pupil characteristics’, and sustainability of the programme. The interview guide was not piloted before use. The duration of the interviews ranged from 43 to 65 min.

The second set of interviews involved the Marathon Champion (i.e. a member of staff) from each of the consenting schools. Due to the geographical spread of these schools, the vast majority were conducted via telephone (*N* = 18) with the remaining (*N* = 3) conducted face to face. Before each interview, information on the aim of the study, approximate duration of the interview and assurance of anonymity and confidentiality were provided. The importance of participants’ own opinions, experiences and ideas were emphasised.

Concepts from the Diffusion of Innovation Theory [29] were used to inform the interview guide and to explore the processes and factors influencing the spread and adoption of the programme. Roger’s theory has been successfully applied to previous school-based implementation studies to provide insight into, and an explanation of, influences on implementation and identify the mechanisms for change [22, 30, 31]. Questions centred around: the process of engaging with MK; influences on the school’s decision to engage with the programme; the delivery in school; facilitators and barriers to implementation; and key learning and recommendations for future development. Probes and follow-up questions were used to encourage participants to talk, provide examples and elaborate on their ideas and opinions. The duration of the interviews ranged from 23 to 38 min.

Following both sets of interviews, the interviewees were given the opportunity to review the written transcripts to clarify or provide further insight. Such use of respondent verification reduced the chance for misinterpretation of results and potential bias and ensured a good understanding of participants’ views [32].

### Focus groups

Once recruited, all Marathon Champions were asked if they would organise at least one focus group with a sample of their pupils. Schools were purposefully selected based on their proximity to the researcher’s workplace. Guidelines were provided to the teachers when selecting pupils, such as; ensuring a range in relation to age, sex, enthusiasm and participation level in the programme, as well as in the pupil’s ability to communicate their experiences and opinions. Pupils were aged between six and 10 years of age, and as suggested by Kennedy et al. [33], focus groups were conducted with groups of between five to eight pupils at a time, grouped by Key Stage^1^. The focus groups took place within the pupils’ respective schools and at a time deemed by the teachers to be the least disruptive to the school day.

Questions focused on pupils’ understanding of physical activity and health, participation in physical activity during school and outside of school, and their participation in MK and use of the programme materials. To facilitate discussion and to ensure pupils understood what physical activity was, the interviewer read out a description of the concept and as an illustration, gave participants photographs of different types of physical activities as well as some of the MK branded tools and resources. The duration of the focus groups ranged from 37 to 70 min.

### Data analyses

All interviews and focus groups were recorded with a digital recorder and transcribed verbatim into Microsoft Word (Microsoft, Redmond, WA, USA) before being imported into NVivo (QSR Version 10.0) to manage and organise the data. Deductive thematic analysis was used to provide a nuanced and detailed account of participants’ views of the facilitators and barriers to adoption and implementation of MK; guided by a priori themes based on Diffusion of Innovation Theory and as identified in the literature [34]. Therefore, an apriori coding manual was not required.

Following Braun and Clarke’s [35] six phases of thematic analysis, the transcripts were firstly reviewed to become familiar with the breadth and depth of content of the data and to generate initial ideas and notes for coding. Data were then coded before collating into identified themes and organising into a thematic map. The candidate themes were subsequently revised and refined to ensure they reflected the meaning evident in the data set, before being named.

The trustworthiness of the findings was facilitated by two methods. Firstly, investigator triangulation processes involved two of the authors independently checking the initial coding strategies and the coding framework generated by the first author. Interpretations were openly discussed and challenged appropriately to achieve a final consensus. At this end stage, the remaining authors then served in the capacity of peer-debriefers or ‘critical friends’ (Cresswell, 1998) by reviewing the framework as a whole and critically probing for explanations of certain decisions made by the first author.

## Results

Twenty-three, of the 54 eligible schools invited to participate, consented; 28 did not respond, and five declined. However, three sets of interviews could not be completed as arranged meaning that a total of 20 interviews were conducted, representing a reach of 37%. The 20 schools from which the participants were drawn included schools of varying status (voluntary controlled, voluntary aided, community, academy). Schools ranged in size (44–718 pupils), geographic location, free school meal eligibility deprivation scores (5.3–51.9% (mean = 19.5%)) and in overall school effectiveness as determined by Ofsted (Inadequate – Outstanding). See Table 1 for a summary of the schools’ characteristics.

**Table 1:** School Characteristics

School	Region	Urban/Rural Description^a^	Size of pupil population^a^	Accessible to all pupils
1	South East	Rural	92	Yes
2	South East	Rural hamlet and isolated	96	Yes
3^a^	South	Rural hamlet and isolated	111	Yes
4	East	Rural town and fringe	176	Yes
5	East	Urban city and town	181	No
6	West Midlands	Rural town and fringe	197	Yes
7	London	Urban major conurbation	120	Yes
8	East Midlands	Urban city and town	165	Yes
9^a^	West Midlands	Rural village	168	No
10^a^	East Midlands	Rural town and fringe	195	Yes
11	London	Urban major conurbation	510	Yes
12	London	Urban major conurbation	198	Yes
13	South	Rural village	58	Yes
14^a^	West Midlands	Urban city and town	200	Yes
15	West Midlands	Rural town and fringe	85	Yes
16	London	Urban major conurbation	600	No
17^a^	East	Rural hamlet and isolated	44	Yes
18	East	Urban city and town	578	No
19	West Midlands	Urban major conurbation	718	Yes
20	London	Urban major conurbation	285	No

Data retrieved from the Ofsted data dashboard

Numbered ^a^ denotes schools that participated in the pupil focus groups

The 20 Marathon Champions included two Head teachers, one Deputy Head teacher, nine PE Co-ordinators, five classroom teachers, two learning support mentors, one teaching assistant and one external sports provider. Eleven of the participants were female, and nine were male, and they ranged from having one to 10 years of teaching experience in their current school at the time of interview.

All schools had implemented MK for a minimum of one academic year. At the time of conducting the interviews, one school was in its third year of implementation, three schools were in their second year, and two schools had ceased implementing the programme after the one academic year.

Nine semi-structured focus groups were conducted with 55 pupils from five of the schools. Participants included 27 girls and 28 boys from across Key Stages 1 and 2 (five pupils from Year 1, seven pupils from Year 2, six pupils from Year 3, seven pupils from Year 4, 18 pupils from Year 5 and 12 pupils from Year 6).

### Barriers and facilitators

Multiple factors were perceived to act as both facilitators and barriers to the implementation of MK and centred around three principal themes corresponding to features and outcome of the programme, implementation decisions and school climate.

### Features and outcomes of the programme

Participants discussed three key areas within this overarching theme, which were believed to facilitate implementation. These sub-themes are ethos; resource; and outcomes.

#### The ethos of the running programme

The ethos of the programme was frequently referred to, particularly in relation to the schools’ decision to adopt the initiative, where MK was felt to be compatible with the school’s values, ambitions, policies and needs. Increasing pupil participation during the school day was a driver for all schools, and many Marathon Champions commented on actively seeking programmes which would add value to their existing provision and appeal to a broad demographic of pupils. In addition, the distinction of the programme as offering an additional opportunity to be active as part of the school day and outside of PE lessons was identified as an important facilitator and potential reason for the high rates of pupil uptake experienced within the schools. It was suggested that this was also related to the inclusive nature of the programme, where pupils exercised autonomy over the frequency, duration and mode of their participation which therefore made it appealing to those pupils who were otherwise disengaged from more formal activity. Teachers and pupils proposed that this aspect of the programme contributed to the pupil outcomes, amongst others, of motivation, enjoyment and sense of achievement, as one Head teacher commented:

I think what I liked and still do like about it, is that it is accessible to all; you don’t need to bring a change of kit to school which is sometimes a barrier. It is something that they can dip in and out of, they don’t have to commit in terms of saying ‘I’m going to run the every week’ you know with lots of clubs that run at school you have to attend them every week because either they are paid for, or we need that level of commitment otherwise the club can’t run. Well this, it’s not like that, if one week the child wants to run they can, or if they want to run for part of their lunchtime they can and then if another week they’re playing a playground game or playing with their friends there’s no expectation that they have to run every week. (Head teacher, School 6).

#### Programme resources

A consistent finding was that the programme resources were useful for monitoring and rewarding pupils’ participation, both in the short term via the lap bands and longer term via the DTS. Furthermore, it was believed that this was reinforced by framing participation in terms of developing a personal best, as one teacher reflected:

Some children have just gained a bit more motivation to have a go and have realised that it is not about winning or losing but about having your own goal and trying to achieve your own goal rather than competing against everybody else. (Key Stage 1 lead, School 1).

This was important for recognising individual effort, especially of those who did not traditionally engage in PE, as well as for providing a lever for teachers to try to maintain pupil enthusiasm and interest. Consequently, many teachers typically remarked how they felt that the programme was important in building the school’s capacity to increase participation.

#### Outcomes

Teachers and pupils discussed the numerous beneficial effects of the programme, particularly for pupils, for example, the fact that MK was perceived as a healthy activity, contributed to improvements in fitness, was a fun activity and provided a sense of personal achievement. However, there was also an acknowledgement of the potential for unintended outcomes of the programme such as pupils compensating for increased activity during the school day with more sedentary pursuits during their leisure time.

### Programme implementation decisions

This theme relates to the school’s decisions around the running programme implementation, that is, judgements relating to the process of delivering the programme.

The decision to employ additional efforts to promote the programme appears to be important. One PE co-ordinator remarked how it had become routine “We just followed the recommended model and have been since September. It really has run itself, it’s become self-perpetuating, and it’s a great club to run.” (PE co-ordinator, School 4). That said, many teachers identified the ability to maintain momentum as a challenge, particularly, as one Head teacher commented, given the duration of the programme over the length of the academic year:

“I think what we found, and we still do find, at the very start loads of children take part and then children drop off a bit and it’s finding ways to refresh it. We sort of put a display up in school to try and promote running as well so the children can see and it reminds them that every Tuesday lunchtime is Kids Marathon day so it’s constantly trying to find ways to refresh it with the children because they do sometimes forget.” (Head teacher, School 6).

The decision as to who would lead the running programme was raised by nearly all schools. Half of the schools followed the recommended delivery model of using MK as a lunchtime activity once or twice a week, designating a specific year group to a certain day, or allowing anyone who wanted to participate an opportunity to accumulate laps. Rather than placing the onus on one member of staff, a few schools opted for individual class teachers to take responsibility for their own class’s participation and used the programme during curriculum time. The reasoning given by one PE co-ordinator was “We chose to do the curriculum because that was easier and simpler for us to cope with as a school” (PE co-ordinator, School 14). This provided teachers with the flexibility to use the programme when they felt most appropriate or practical and depending on what else was happening in school that week.

While this flexibility was welcomed by the majority of teachers; they also indicated that there could be substantial variation in the frequency pupils, of different classes in the same school, accessed the route and were able to complete their laps. Consequently, a hybrid of different tried and tested approaches to implementing MK was reported, as revealed by one teacher:

Again, I don’t stick to anything; I tend to fit it in when I can. Sometimes I think right yes we can. Because of the way the classroom runs we sort of do morning activities first thing in the morning so sometimes I think right put that into my morning activity, sometimes after play I’ll say let’s stay out and do it, sometimes we might finish the lesson at 12 o’clock and we don’t have lunch until 12.15 we might go out then. It is very much when I’ve got 10, 15 min in the timetable (Deputy Head teacher, School 17).

As recommended to the schools at the launch, many schools also made use of their young leaders and sports ambassadors to assist with the administration of the programme, including the distribution of lap bands during the running and the recording and monitoring of data. This was particularly important for those schools which had designated days for different year groups to participate and where there was still a need for lunchtime staff to supervise those pupils who were having lunch and/or not participating. One teacher commented that “It was not staffed by staff because it wasn’t their job and they had other stuff to do” (Senior learning mentor, School 19).

The decision to change elements of the programme (flexibility) was a notable point raised by teachers. To ensure all pupils could achieve the marathon distance by the end of the year, each school was provided with a recommended number of weekly laps for pupils to complete (this was based on the distance of their route and the number of weeks available in the school term). In addition, where space permitted, KRF measured and marked two running routes on the school grounds (i.e. one on the playground and one on a grass area) to increase the likelihood of pupils being able to use the programme in the event that one of the routes was unavailable due to other activities or inclement weather. Some schools also adopted a flexible policy on how pupils accumulated their laps by allowing distances completed outside of school, verified by a parent or guardian, to contribute to their marathon distance (e.g. a measured run or walk, such as Parkrun and sports matches) being ‘converted’ to recognise extra-curricular individual participation. The benefits of this approach were highlighted by one Head teacher: “he does everything he can to avoid doing it at school, but it’s bringing in celebrating what he does out of school.” (Head teacher, School 3).

However, a few teachers were equally aware of over exploiting the flexibility of the programme, to the point where the impact may be diluted with the sense of structure and focus becoming lost. This was particularly important for pupils, one of whom reported frustration and confusion over the lack of communication and/or inconsistent use of such adaptations affecting their ability to plan and track their own participation:

“Well I just sometimes got fed up because at that time I was quite close to finishing my Marathon and I really wanted to finish it but then sometimes they wouldn’t let us do it and I couldn’t really finish it quickly.” (Year 5 Boy, School 3).

It became clear that the decision to use other methods to track individuals were utilised by schools**.** Teachers and pupils reported a variety of methods used to track pupils’ progress towards achieving the marathon distance. Typically, administration of lap bands was completed as part of afternoon registration by individual class teachers and entered into the DTS by the Champion on a weekly basis. Some Marathon Champions made use of the tools (e.g. the wall chart) provided as part of the programme to feedback to pupils, as one explained: “So they will all have some sort of total on there which we will communicate to them.” (Teacher, school 8), whereas for others, this was more infrequent and prompted by the return of the DTS to KRF every half term. Pupils’ participation was consistently rewarded using the stickers and certificates provided. Some teachers spoke of linking these to their school’s reward system by awarding house points for key milestones; however, others felt that rewarding pupils based on outcome was limiting and also incorporated certificates to reward and reinforce the notion of personal improvement (e.g. for most improved or most consistent participation). School celebration assemblies were also frequently used to award the certificates and engage parents in celebrating success. A couple of the schools also used assemblies to present the data, as one Champion shared:

I thought we’d tally up each week to see how far they had run and we ran to Greece or we ran to Oslo or wherever. I added that little bit you know within assembly, I put the map on the big screen and we said right this week we’ve run this far and I plotted it on the map. (Learning support assistant, School 20).

### School climate

Participants discussed four key areas relating to the physical, social and educational dimensions of the school and these are presented as the sub-themes of whole school engagement, school culture; physical environment; and PE and physical activity policies and goals.

#### Whole school engagement

Engagement (i.e. participation in organising delivery of the programme or positive support towards the delivery of the programme) was the most prominent sub-theme, in terms of the strength of feeling and frequency of references associated with it. The importance of support from senior management and staff buy-in was mentioned by all teachers, with one adding: “I think probably the Head has to take it on as a concept and then delegate it.” (Learning support assistant, School 20). The decision to adopt was always made at a strategic level, by or in association with senior management, and aligning the programme with the vision and aims of the school, as one Head teacher described:

I think it fits well because we value all parts of our child even though we have to assess the academic ones, I think all parts of our holistic development, our mental emotional and physical state is important too. So I think it fits well with that, it’s encouraging the children out, it’s encouraging them to move and to value their physical health which I think is important. (Deputy Head teacher, School 13).

Staff buy-in and engagement was also perceived as particularly important in securing commitment to adopt the programme but also essential in ensuring its efficient administration, as for example, the recording of weekly laps most frequently took place in the classroom as part of the afternoon registration process. This, in turn, facilitated the reinforcement of messages about the programme as teachers were able to provide feedback to pupils on their progress. On this, one teacher commented:

I always entered my class’ with my class so they could see, they could actually see their progress and we got lots of maths out of it then, seeing how many more laps I would need to get to and do I think other teachers started to do that as well because I think that really started to help motivate them rather than them running their laps and not really finding anything out about it. (Key Stage 1 Lead, School 1).

For meaningful impact, some teachers commented that a programme of this nature needed an advocate with sufficient influence and autonomy to galvanise the whole school and secure commitment from staff, something which was also mentioned by the programme team: “The person needs to have charisma because as a result of that people listen to them, the head listens to them, the children listen to them.” (CEO, Kids Run Free). Responsibility for the delivery of MK typically lay with the member of staff who led on the delivery and co-ordination of PE. However, the majority of these people, while having an interest in, or participating in, running themselves, were not a specialist in PE. Furthermore, the PE co-ordinator role was always taken on in addition to the individual’s other roles and responsibilities for teaching; unsurprisingly, therefore, these staff members capacity to deliver MK was one of the most frequently cited barriers to implementation. One PE teacher reflected on how this influenced the decision to adopt the programme:

One of the concerns within school at the moment is how much time it is going to take up as a starter and manage and how much admin is there and what it’s going to cost. So those are the kind of key pressures that teachers in schools are under so I had to clarify those before I could get permission from the head to go and then do it. (PE teacher, School 12).

While Head teachers were identified as being integral to the long-term implementation of the programme, the Champions were responsible for the schools’ organisation and administration of resources to deliver it. However, Champion engagement varied significantly between schools, ranging from being a point of contact for KRF, taking responsibility for maintaining the monitoring and recording of data, to supervising the pupils during the running sessions and acting as an advocate for the programme in an attempt to maintain pupils’ and staff interest and enthusiasm, as reflected in one PE co-ordinator’s experience:

My involvement is, in that regard, quite limited in that every teacher is responsible for their class which actually makes it a lot easier for me because I’ve sort of delegated it out. It’s easier for every teacher to be in charge of their class, then at the end I’m the one that just goes round and makes sure that everyone is sorting it all out. (PE co-ordinator, School 14).

While staff capacity and the ability to respond to competing demands on teachers’ time was identified as a significant limiting factor impacting implementation, the majority of teachers anticipated a certain level of time and commitment required to administer the programme. Importantly, this was not perceived to be atypical of such an initiative or overly burdensome.

#### School culture

The norms, values and beliefs of the school were identified as a sub-theme. In particular, the social aspect of the programme was particularly crucial for pupils, who reported peers to be the single most significant influence on their participation. This was closely linked to a sense of competition which was inspired by the programme and operated at different levels. For example, competition with self and development of a ‘personal best’ were reported by many pupils as being a facilitator, particularly those less engaged in traditional activities. Others interpreted the programme as competition with their peers and used the programme to reinforce their sense of identity as being ‘more sporty’. On occasion, the programme was also purposefully used by the Champions to foster a sense of competition as part of the school’s climate, as one teacher explained:

The drive of children and the ethos in the school is quite competitive now, which is something that I wanted to get back into school and my parents have commented on. Having that competitive nature and the chance for children to experience different things, I think it fits really well within our school (PE Co-ordinator, School 9).

Many teachers also commented on the sense of cohesion across the school, especially in schools where pupils from multiple year groups participated concurrently. One teacher added how the programme had given them an opportunity to do something together. “I think it’s been good for building relationships amongst the pupils.” (PE Co-ordinator, School 10). Furthermore, teachers and pupils also mentioned how they thought their participation in the programme was important in developing the school’s profile and reputation and that the school would be perceived more favourably by others, such as parents and the wider school community, as a result of taking part. This was particularly true for one Champion who said:

For us a small school, we can’t get together a netball team and a second team or whatever and you go to these festivals of things and people have got their 1st team, their 2nd team well were’ scrabbling together to make a team so we never win anything. We’re always just a laugh, and the kids generally accept that, but with the running we’re not going to be last (Deputy Head, School 17).

#### Physical environment

Regarding the physical environment, the size of outdoor space available for the programme was identified as a challenge in some schools. This was particularly so for the larger ones who catered for more pupils and were not able to dedicate sole use of a particular area to one activity, or those in which wet weather policy did not permit the use of the route in inclement weather. Equally, in others, it was felt that the programme encouraged the use of existing facilities and space by adding value to current provision to attract different types of pupils. This was true for one teacher who said:

Running was one of the things that they’d always done in school, because of our location, but it hadn’t been done in any sort of organised way. It was a bit ad hoc, the teacher would just take them out for a run. So the Kids Marathon seemed a really good way of getting the children engaged again in running. (Deputy Head teacher, School 17).

#### PE and physical activity policies and goals

Existing PE strategies and approaches to promoting and delivering physical activity (and the extent to which MK aligned with them) were identified by the majority of schools as being important to implementation, not only regarding the decision to adopt the programme but also for its day to day management. For example, several of the teachers commented on the use of the programme to boost formal activity opportunities outside of the curriculum, to target specific groups of pupils and/or to contribute to the school’s application for accreditation and/or award schemes.

## Discussion

This study aimed to better understand the barriers and facilitators to the implementation of a running programme, Marathon Kids, within primary schools in England. It found that there was variability in programme implementation across different schools and that multiple factors were reported to act as both facilitators and barriers, most of which related to the physical, social and educational dimensions of the school e.g. culture, whole school engagement, PE and physical activity policies and goals and the physical environment.

These findings are consistent with previous studies examining factors influencing the implementation of school-based physical activity programmes. For example, contextual factors and programme factors have been identified as being influential for programme implementation in other studies [36, 34]. Similarly, studies examining factors related to the implementation of school-based physical activity specifically have also identified delivery system characteristics (i.e. a supportive school climate) to be influential [22, 34]. This study similarly confirms the importance of these factors for a school-based running programme and suggests that they are critical features to consider when designing school-based physical activity interventions.

Staff ‘buy-in’ was consistently identified as the main barrier for schools when implementing MK. Teachers and school staff have a significant and influential role in the implementation of external programmes in schools and this was recognised by participants in the present study. Teachers’ attitudes concerning the intervention, i.e. whether they thought it worthwhile and whether they liked it or not, have been found to be associated with the level of adherence to health promotion programmes in schools [37]. Therefore, interventions should consider what training and or support could be given before implementation to ensure commitment and participation by pupils and teachers alike. Engaging the whole school community to support physical activity opportunities by training teaching support staff and volunteers such as pupil peer leaders or young ambassadors may be a viable option to strengthen the capacity in schools where specialist knowledge or expertise is not necessary for such informal activity.

It might be useful to explore more automated systems of tracking and monitoring participation in the programme. Technology such as radio frequency identification (RFID) systems are increasingly being used as an objective assessment of children’s movement patterns during physical activity [38] and may serve as a useful administrative and pedagogical tool in assisting with the data collection administration and/or allowing such data to be incorporated into/analysed in lessons. There were some teachers who did report using the programme as a context for learning (e.g. using the data to explore the use of decimal places) suggesting that there is potential for associated learning which would make it more ‘educational’ and suitable for a school-based programme, particularly as part of a cross-curricula whole school approach.

Interestingly, many of the themes identified were reported to act as both facilitators and barriers across the different schools (e.g. school environment, pupil choice and control, peer influence and interrelationships). These identified issues should be areas of focus for programme developers as well as school staff to ensure that strategies are available to mitigate these factors when they serve as barriers, in an effort to support programme implementation. The implementation process has previously been described as an interrelated set of sub-processes progressing simultaneously at multiple levels within an organisation [39]. This was evident in the current study whereby both staff and pupils also described the interrelated nature of many implementation factors. For example, teachers stated that allocating individual teachers responsibility for their own class’ participation, led to those involved being more knowledgeable about pupils’ progress and more likely to use the tools and resources to provide feedback and rewards. Additionally, participants described these factors in a way which suggests that they commonly operate in a sequential and sometimes cyclic manner but generally acted in the same direction depending on whether they were perceived to be a barrier or a facilitator. For example, some pupils talked about how the ad hoc use of the programme restricted their opportunity to participate because their ability to plan their participation was compromised. These pupils subsequently became demotivated due to the inequitable access to the programme in comparison to their peers and the perception that they were less likely to achieve the marathon challenge.

Due to the interrelatedness of the themes, it is clear that there is no ‘one size fits all’ approach when it comes to implementation and that while programmes need to offer a degree of flexibility, there is a need to balance schools’ autonomy to adapt delivery with the contextual diversity of the different schools. There was an acknowledgement (and almost expectation) among teachers that different schools would be implementing the programme in different ways and that this may not always reflect the intended delivery model. Indeed, there were numerous examples of teachers adopting a flexible policy on how pupils accumulated their laps. Such modifications are important to consider regarding whether they may strengthen programme outcomes or undermine the programme’s aims and objectives and participant experience. Rigidity in implementation has previously been identified as a characteristic of an ineffective implementer within schools [13], therefore, local adaptations may be necessary and as in this case, the contribution of pupils’ other physical activities to accumulated distance did not appear to reduce the amount of physical activity pupils engaged in, but conversely motivated some to be more active and engage in other activities outside of the school setting. This is particularly pertinent given the importance of long-term participation in physical activity as part of a healthy lifestyle.

Running programmes offer a practical approach to providing additional physical activity time outside of PE [40] without the need for extra playground equipment or specialist teachers. However, for physical activity programmes to be successful, it has been suggested they need to become embedded within the culture of the school, that is, shift from an individual behaviour orientation towards more of a sociological approach, focussing on the interaction between the school, teachers and individual pupils [41]. This was apparent in one school where the Marathon Champion purposefully set out to implement the programme integrating it into aspects of teaching and learning, e.g. through PE and Maths, strengthened partnerships both internally and externally to the school as part of the whole school community e.g. by using young leaders, engaging parents and sharing experiences with colleagues from the borough sports partnership, and developed the culture and ethos of the school where physical activity was valued and supported and given a high profile, e.g. via a MK display board, updates in the school newsletter and a marathon themed fundraising event. It may be that such programmes would be well placed as part of a broader framework of health promotion such as a comprehensive whole school approach to physical activity [42].

### Strengths and limitations

This study is unique in being, to the authors’ knowledge, the first to examine perceptions of barriers and facilitators influencing the implementation of a school-based running programme in the UK. Moreover, it does so from a number of different stakeholders, including programme developers and deliverers and teachers and pupils from a diverse range of schools, thus adding to the limited evidence base on teachers’ and pupils’ perceptions of physical activity programmes in schools.

As is the nature of qualitative work, it should be noted that these findings cannot be generalised to all primary schools. However, a diverse range of schools in terms of type, size and demographic, were included and thus the authors believe the data presented has implications for similar schools in the UK.

Schools volunteered to take part in the study and interviews were reliant on self-report responses concerning implementation. This may have resulted in a selection bias with recruited participants having a greater interest in the programme and more likely to have had a positive experience implementing it. However, even in this group of participants, several barriers were apparent that could also apply to less motivated school staff e.g. inclement weather and limited outdoor facilities/space. A further limitation was the purposive selection of focus groups based on proximity to the researcher’s workplace which may have introduced selection bias.

### Implications for practice

Given the current popularity of school-based running programmes within the UK, this research is timely in helping to contribute to understanding on the implementation of running specific programmes and school-based physical activity programmes more broadly. The findings can be used to guide practice by encouraging practitioners and school staff to consider what steps could be taken to facilitate implementation, particularly before delivery in school. If initiatives provided by teachers are to be successful in changing pupils’ behaviour and increasing physical activity levels, there are a number of factors which should be taken into consideration relating to the school’s readiness to implement. These are often ignored and/or given insufficient attention from the outset making implementation more challenging and positive/sustained outcomes less likely [43] and may be related to the range of individuals within the school who will support and/or be directly involved, such as the Head teacher, the staff and pupils, as well as how the programme might best be adapted to suit the local context, meet pupils’ needs, and complement existing educational and school-based initiatives, policies and practices. Practical recommendations for schools are provided in Additional file 3. The recommendations have been developed in response to the experiences and feedback received from conducting the interviews, and are deemed by the Champions to be useful considerations when engaging with a physical activity programme.

## Conclusion

The findings from this study suggest that barriers and facilitators to programme implementation are varied and span programme, organisational and system-level factors. Furthermore, many of these implementation factors are interrelated and commonly operate in a sequential and sometimes cyclic manner but generally acted in the same direction depending on whether they were perceived to be a barrier or a facilitator.

As interest around school-based running programmes continues to grow, understanding the type of factors which can influence implementation to maximise and sustain uptake is essential. This study has identified implementation areas to consider both for programme design and evaluators working within these programmatic contexts. Future research would benefit from focusing on how best to equip schools for effective implementation of physical activity programmes. For example, by supporting communication and building capacity in providing additional physical activity opportunities for pupils.

## Additional files

Additional file 1: Standrads for reporting qualittaive research checklist. (DOCX 21 kb)

Additional file 2: Interview/Focus Group Topic Guide Excerpts. (DOCX 20 kb)

Additional file 3: **Table S2.** Practical recommendations for schools to facilitate the implementation of school-based physical activity programmes. Specific recommendations and implications for practice when implementing a school-based running programme. (DOCX 17 kb)

## Abbreviations

DTS: Digital tracking system
KRF: Kids Run Free
MK: Marathon Kids
PE: Physical education
RFID: Radio frequency identification

## Footnotes

1 A Key Stage is a stage of the state education system in England, Wales and Northern Ireland setting the educational knowledge expected of students at various ages. In primary schools, these include Key Stage 1, consisting of children in Years 1, 2 and 3 (children of five to seven years of age) and Key Stage 2, consisting of Years 4, 5 and 6 (children eight to11 years of age).
