# Supplementary material for: A retrospective qualitative evaluation of barriers and facilitators to the implementation of a school-based running programme
Source: BMC Public Health. 2018 Oct 20;18:1189. doi: 10.1186/s12889-018-6078-1 (PMC6196020; doi:10.1186/s12889-018-6078-1)
Supplement: Supplementary file 2 — Interview/Focus Group Topic Guide Excerpts. (DOCX 20 kb) [file 12889_2018_6078_MOESM2_ESM.docx]

**Additional File 2: Interview/Focus Group Topic Guide Excerpts**

**Kids Run Free interview**

***Knowledge and expertise for implementation***

- Typically who is the main member of staff responsible for facilitating Marathon Kids in school? What is their role?
- What type of familiarisation/training is needed and for whom, for schools to successfully use KM?
- Is written information/instructions available for school staff? In what format?
- How necessary is it for other staff members/the wider school community to have knowledge of Marathon Kids?
- Is direct assistance from KRF needed to successfully implement the programme? What is the added value?
- What would you describe as the attributes for a successful school based implementer?

**Marathon Champion Interview**

***Key learning and recommendations for future development***

- With the benefit of hindsight, what if anything, would need to be done differently to better implement Marathon Kids in to your school?
- What positive or negative impact (if any) do you think the programme had on;
  - the pupils
  - the staff
  - the wider school community
- Is Marathon Kids *currently* running in your school? If so, how often and in what way? If not, when and why did it stop?
- Could the programme be made more accessible/attractive to a school in order to engage? If so, in what ways? What would need to change
- Do you/would you recommend Marathon Kids to other schools? If so, why
- What advice would you give a school contemplating taking on the Marathon Kids project?

**Focus Group**

***Views on participating in Marathon Kids***

- How would you describe Marathon Kids to me?
- What do you think you learnt from the assembly presentation?
- Did you take part in Marathon Kids?
  - If yes, how often did you do it? When did you do Marathon Kids (e.g. break time, lunch time)?
- What did you like or dislike about taking part in Marathon Kids?
- Do you think participating in Marathon Kids has changed your, or your friends, physical activity, if so how?

Pupils will be shown pictures of children taking part in Marathon Kids project as well as the various branded project materials e.g. pupil diary, class poster, wristbands, stickers, route markers, certificates, medals etc. Each picture will be placed on the table one at a time and the researcher(s) will provide a brief verbal summary of what the picture includes. When familiar with the pictures pupils will be asked the following questions:

- Which of these things do you remember seeing or using as part of Marathon Kids?
- How did you use them?
- What did you like or dislike about them?
- How could the Marathon Kids be changed to make it better for you and/or your friends?
