# Supplementary material for: A retrospective qualitative evaluation of barriers and facilitators to the implementation of a school-based running programme
Source: BMC Public Health. 2018 Oct 20;18:1189. doi: 10.1186/s12889-018-6078-1 (PMC6196020; doi:10.1186/s12889-018-6078-1)
Supplement: Supplementary file 3 — Table S2. Practical recommendations for schools to facilitate the implementation of school-based physical activity programmes. Specific recommendations and implications for practice when implementing a school-based running programme. (DOCX 17 kb) [file 12889_2018_6078_MOESM3_ESM.docx]

Table 2 Practical recommendations for schools to facilitate the implementation of school-based physical activity programmes

| **Stage of Implementation** | **Practical strategy to facilitate implementation** |
| --- | --- |
| Pre-Implementation | - Conduct a brief consultation with staff, pupils and possibly parents to identify interest in a running programme and any potential barriers plus strategies to negate or minimise their impact. - Identify a member of school staff who can fulfil the role of programme Champion within school. The success of this role is more dependent on credibility, influence, willingness and capacity, rather than seniority and an interest in physical activity is desirable but not essential. - Establish the type of support available to help the Champion deliver the programme. For example, the use of young leaders and peer champions can be very effective and help to engage pupils and sustain their participation. - Identify clear, school specific, aims and priorities for the programme including what success means for the school and how the programme could be sustained and embedded into practice. In doing so it is important to also identify any potential unintended outcomes e.g. a compensatory reduction in attendance at after school clubs. - Consider how the programme will interact with current practice and interests within the school e.g. by complementing or reinforcing existing practice and/or driving change. |
| Initial Implementation | - Where possible, secure the tangible support of senior members of staff e.g. by encouraging their participation in the activity itself or visibility in the playground while the programme is underway. This is particularly useful during early delivery but can also be beneficial in sustaining motivation for, and interest in, participation. - Make sure the multiple benefits of participating in the programme are communicated and reinforced to pupils, with a particular focus on the more immediate short-term benefits e.g. fun, opportunity to get some fresh air and spend time with friends. |
| Adaptation and evolution | - Engage with pupils to review the programme on an ongoing basis to ensure it continues to meet their developmental needs, and adapt and tailor it accordingly. Ensure that any adaptations are communicated clearly to pupils and are applied consistently and monitored in order to limit the amount of unwarranted adaptation. - Evaluate the delivery of the programme and what impact it is having e.g. in line with the agreed programme aims for the school. Discuss possible adaptations with other members of staff within the school and the programme organisers/ support team. |
